# *Aurelia aurita *(Cnidaria) Oocytes' Contact Plate Structure and Development

**DOI:** 10.1371/journal.pone.0046542

**Published:** 2012-11-21

**Authors:** Leonid S. Adonin, Tatyana G. Shaposhnikova, Olga Podgornaya

**Affiliations:** 1 Institute of Cytology RAS, St. Petersburg, Russia; 2 Department of Cytology and Histology, Faculty of Biology and Soil Sciences, St-Petersburg State University, St. Petersburg, Russia; Technische Universität Dresden, Germany

## Abstract

One of the *A. aurita* medusa main mesoglea polypeptides, mesoglein, has been described previously. Mesoglein belongs to ZP-domain protein family and therefore we focused on *A.aurita* oogenesis. Antibodies against mesoglein (AB RA47) stain the plate in the place where germinal epithelium contacts oocyte on the paraffin sections. According to its position, we named the structure found the “contact plate”. Our main instrument was AB against mesoglein. ZP-domain occupies about half of the whole amino acid sequence of the mesoglein. Immunoblot after SDS-PAGE and AU-PAGE reveals two charged and high *M_r_* bands among the female gonad germinal epithelium polypeptides. One of the gonads' polypeptides *M_r_* corresponds to that of mesogleal cells, the other ones' *M_r_* is higher. The morphological description of contact plate formation is the subject of the current work. Two types of AB RA47 positive granules were observed during progressive oogenesis stages. Granules form the contact plate in mature oocyte. Contact plate of *A.aurita* oocyte marks its animal pole and resembles Zona Pellucida by the following features: (1) it attracts spermatozoids; (2) the material of the contact plate is synthesized by oocyte and stored in granules; (3) these granules and the contact plate itself contain ZP domain protein(s); (4) contact plate is an extracellular structure made up of fiber bundles similar to those of conventional Zona Pellucida.

## Introduction

*Aurelia aurita* medusa is the sexual adult stage in a complex animal life cycle. Members of phylum Cnidaria are thought to be diploblastic, possessing only two tissue layers: endoderm (gastroderm) and ectoderm. Medusa possess a huge extracellular matrix (ECM) – mesoglea, between two cell layers. Two types of fibers, which are embedded in a jelly-like substance, have been described at the morphological level in cnidarians mesoglea: collagen-like and the so-called “elastic” or vertical fibers [1], [2], [3], [4]. The main part of *A.aurita* mesoglea is ECM but it also contains a number of mesogleal cells (Mc). The population of Mc inside mesoglea was observed in other species of Scyphozoa and Anthozoa [5]. This feature is not unique but rather rare and little was known about their functions. We determined the polypeptide composition of mesogleal cells (Mc) and mesoglea (Mes) raised antibodies (AB) against one of the major mesogleal protein and checked the antibodies specificity. Using light and electron microscopy immunostaining, we showed that Mc are involved in the formation of mesogleal fibres [6].

The apparent molecular mass (*M_r_*) of polypeptide the AB was raised against is 45/47 kDa (p47). A protein with a similar *M_r_* was observed in the *Hydra* ECM, but it did not react with any AB against known vertebrate ECM proteins, and no suggestion was made about its nature [7]. We supposed that p47 of *A.aurita* mesoglea could be an unknown protein and we made an attempt to clone its gene [8]. The merged sequences acquired by 3′ and 5′ RACE produced mRNA sequence 1421 bp long. An NCBI BLAST [9] search for homologous nucleotide and protein sequences revealed that the mRNA sequence was novel. It was submitted to GenBank (Accession No. DQ467654) and named mesoglein [8].

The search for known domains and motifs in the deduced protein sequence reveals similarity of amino acid positions 43–84 aa to Delta/Serrate/Lag-2 (DSL) domain and similarity of positions 93–337 aa to Zona pellucida (ZP) domain. Mesoglein happens to belong to ZP-domain protein family and therefore we looked more closely at *A.aurita* oogenesis.

The *A.aurita* medusa, widely known as the moon jellyfish, reproduces sexually during summer. Gametes are released from the gonads into the gastric pouches. Ova, produced quickly by oogenesis, remain in the female body but spermatozoa exit via the mouth into the sea [10]. They enter the mouth of a female and make their way to the gonad where they fertilize the eggs. Embryos are released from the mouth and brooded on the oral arms.

AB RA47 against mesoglein stain the plate in the place where germinal epithelium contact oocyte on the paraffin sections. According to its position, we named the structure found the “contact plate”. The description of the morphological formation of *A.aurita* contact plate is the subject of the current work

## Materials and Methods

### Animals

*A.aurita* medusa were collected in the vicinity of the White Sea Biological Station of the Zoological Institute RAS “Kartesh” (Chupa Inlet, Kandalaksha Bay in the White Sea) (http://www.zin.ru/kartesh/default_en.asp) during the summers of 2007–2011.

No specific permits were required for the described field studies; no specific permissions were required for these locations/activities; the location is not privately-owned or protected in any way; and the field studies did not involve endangered or protected species.

The methods of mesoglea (Mes), mesogleal cells (Mc) and ectoderm (Ect) isolation have been published [6].

The female gonads (Gf) were cut from the gastric pocket under a dissecting microscope and were frozen or fixed immediately.

*In vitro* fertilization was done under phase contrast microscope. Eggs were removed mechanically, i.e. pipetted off the germinal epithelium, placed on the slide in sea water and spermatozoids were added. Eggs in the process of fertilization were fixed in 4% paraformaldehyde in PBS for 24 h, then rinsed sequentially in 30%, 50% and 70% ethanol (for 30–40 min each rinse) and DAPI stained after rehydratation. Images were taken by photo camera (Leica).

### Electrophoresis and Immunoblotting

Mes, Mc, Ect and Gf polypeptides were separated by SDS-PAGE [11]. Acid-Urea Polyacrylamide Gel Electrophoresis (AU-PAGE) was used for the charged proteins separation [12], [13]. “Lidase” preparation (Microgen, Perm, Russia), which contains *Bos taurus* hialuronidase (pI 9.2) was used as marker protein. Acrylamide concentration was 10% in the SDS electrophoresis gels and 5% or 7% - in the AU-PAGE gels. Gels were stained with 0.1% Coomassie Brilliant Blue R-250 or G-250.

Polypeptides separated in SDS-PAGE and AU-PAGE were transferred to polyvinylidene difluoride (PVDF, Sigma, USA) membrane or nitrocellulose membrane (Sigma) in a Trans-blot [14] The membranes were blocked with 5% skimmed milk at PBS-Tw for 1 h or overnight. The membrane was then immersed in PBS-Tw with RA47 (1∶5000 dilution) for 1 h, then washed with PBS-Tw three times. Polyclonal antibodies (RA47) against the mesoglein were characterized previously [6]. The secondary antibody commercial stock (GAR-AP, Sigma, USA) was diluted 1/10000 (v∶v) in PBS-Tw. After incubation for 1 h at room temperature with shaking, the membranes were washed twice with PBS-Tw (10 min each). Finally, the sites of enzyme binding were developed with BCIP-NBT. All operations were carried out at room temperature with shaking. Rabbit pre-immune serum instead of RA47 was used as the control and it did not produce any signal.

### Immunofluorescence

Small pieces (2 mm^3^) of Gf from a gastric pocket were fixed in 2% paraformaldehyde in PBS for 24 h and then rinsed sequentially in 30%, 50% and 70% ethanol (for 30–40 min each rinse). The fixed samples were embedded in paraffin blocks to give 5–7 µm thick sections. The sections were preincubated with 5% skimmed milk in TBS-Tw for 1 h, washed 4 times for 10 min with PBS-Tw, incubated with RA47 at 1∶5000 dilution (for 1 h), washed and incubated with Rhodamine-conjugated goat-anti-rabbit Ig (GAR-Rhodamine, Sigma), and visualized with confocal microscope (Leica DM6000). All the procedures were carried out at room temperature (20°C). For controls, some sections were incubated with non-immune rabbit serum followed by the same subsequent steps.

### Transmission Electron Microscopy (TEM)

The pieces of female gonad were fixed in 0.25% glutaraldehyde and 2% paraformaldehyde in PBS (for 1–2 h), postfixed for 1 h in phosphate-buffered 1% osmium tetroxide and dehydrated through a graded series of alcohol and acetone. Finally, the tissues were embedded in Epon 812 (Sigma) according to manufacturer recommendations.

Semi-thin sections were prepared and stained with acid fuchsin according to Geyer [15] and used for preliminary analysis of the material.

***Ultrathin sections*** were cut with a diamond knife with Leica EM UK6, transferred to the support grid (100 mesh, Sigma), stained with uranyl acetate and lead citrate and examined with a transmission microscope (Libra 120, Zeiss).

### Immonogold

The materials were fixed in 2% paraformaldehyde and 0.5% glutaraldehyde in PBS at 10°C for 2 h, washed in 0.05 M ammonium chloride in PBS, and dehydrated before final embedding in LRWhite resin (Fluka) for immunostaining. Non-specific binding was blocked by preincubating the ultrathin sections with 10% bovine serum in TBS-Tw for 1 h. The sections were then incubated for 45 min with RA47 (diluted 1∶400 in TBS-Tw), washed in TBS-Tw, incubated with 10 nm gold-conjugated secondary goat-anti-rabbit antibody (Sigma) for 1 h, washed in distilled water and dried. For controls, some sections were incubated without RA47 (only PBS-Tw) followed by all the subsequent steps. Observation was carried out in transmission microscope (Libra 120, Zeiss).

All images were prepared for publication using Adobe Photoshop CS2 software.

## Results

### Electrophoresis and Immunoblotting

**Figure 1:**
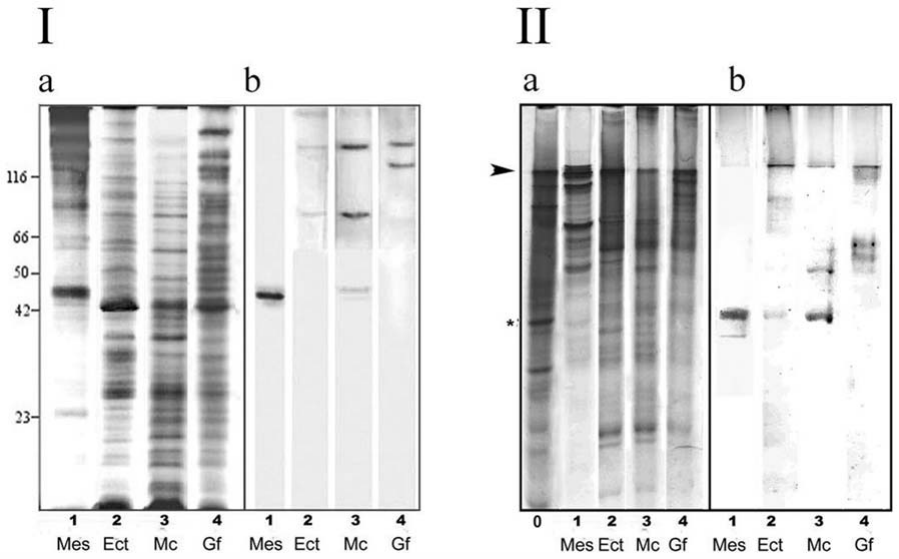
Electrophoresis and immunoblot of medusa cell types. I – SDS-PAGE (a) and immunoblot (b). a – Coomassie-stained 10% SDS-PAGE: 1 (Mes) – mesoglea of adult *A.aurita*; 2 (Ect) – ectodermal tissue layer; 3 (Mc) – mesogleal cells; 4 (Gf) – germinal epithelium of female gonad. ***M_r_*** of marker proteins in kDa are indicated on the left. b – immunoblot of the correspondent lines - Mes, Ect, Mc and Gf. II – AU-PAGE (a) and immunoblot (b). a – Coomassie-stained 7% AU-PAGE: 0 – hialuronidase (pI 9.2) marked with asterisk; 1 (Mes) – mesoglea of adult *A.aurita*; 2 (Ect) – ectodermal tissue layer; 3 (Mc) – mesogleal cells; 4 (Gf) – germinal epithelium of females' gonad. Arrow marks the start of the separating gel. b – immunoblot. For both immunoblots (b) - primary antibodies RA47 in final dilution 1∶5000; secondary antibodies were antirabbit IgG conjugated with alkaline phosphatase in final dilution 1∶20000 (Sigma).

Mesoglea (Mes) contained some cells, but cell proteins of the mature medusa Mes were not detected at the level of one-dimensional SDS-PAGE resolution (Fig. 1, I, 1). The major protein components of mature medusas' Mes were likely to be components of ECM. The main Mes polypeptide was of apparent molecular mass (*M_r_*) 45/47 kDa (p47). – mesoglein. Polyclonal AB raised (RA47 for Rabbit Antibodies) against mesoglein stain p47 itself in Mes preparations. In Mc p47 was a minor stained zone, but the most prominent were zones with higher *M_r_*. The same high zones were stained in the epidermis though they were less prominent. The gastroderm sample did not contain any polypeptides with corresponding antigenic determinants (not shown). We presume that mesoglein is synthesised by cells as a high molecular mass precursor and it undergoes restricted proteolysis during incorporation in ECM. Immunoblot reveals two high *M_r_* bands among the female gonads (Gf) germinal epithelium polypeptides (Fig. 1, I, 4). One of the gonad polypeptides *M_r_* corresponded to that of Mc, the other ones' *M_r_* was higher.

Histochemical characteristics of the Mc implied that its proteins could be highly charged [16]. Mesoglein is charged: on AU-PAGE mesoglein moves from anode to cathode which signifies its high positive charge [8]. The germinal epithelium subjected to immunoblot with RA47 revealed charged proteins (Fig. 1; II). The charge of the proteins, recognized by RA47 in gonads, indicates their relation to mesoglein, though the difference in the electrophoretic mobility could be caused by the difference in charge as well as in *Mr*.

### Immunofluorescence

**Figure 2:**
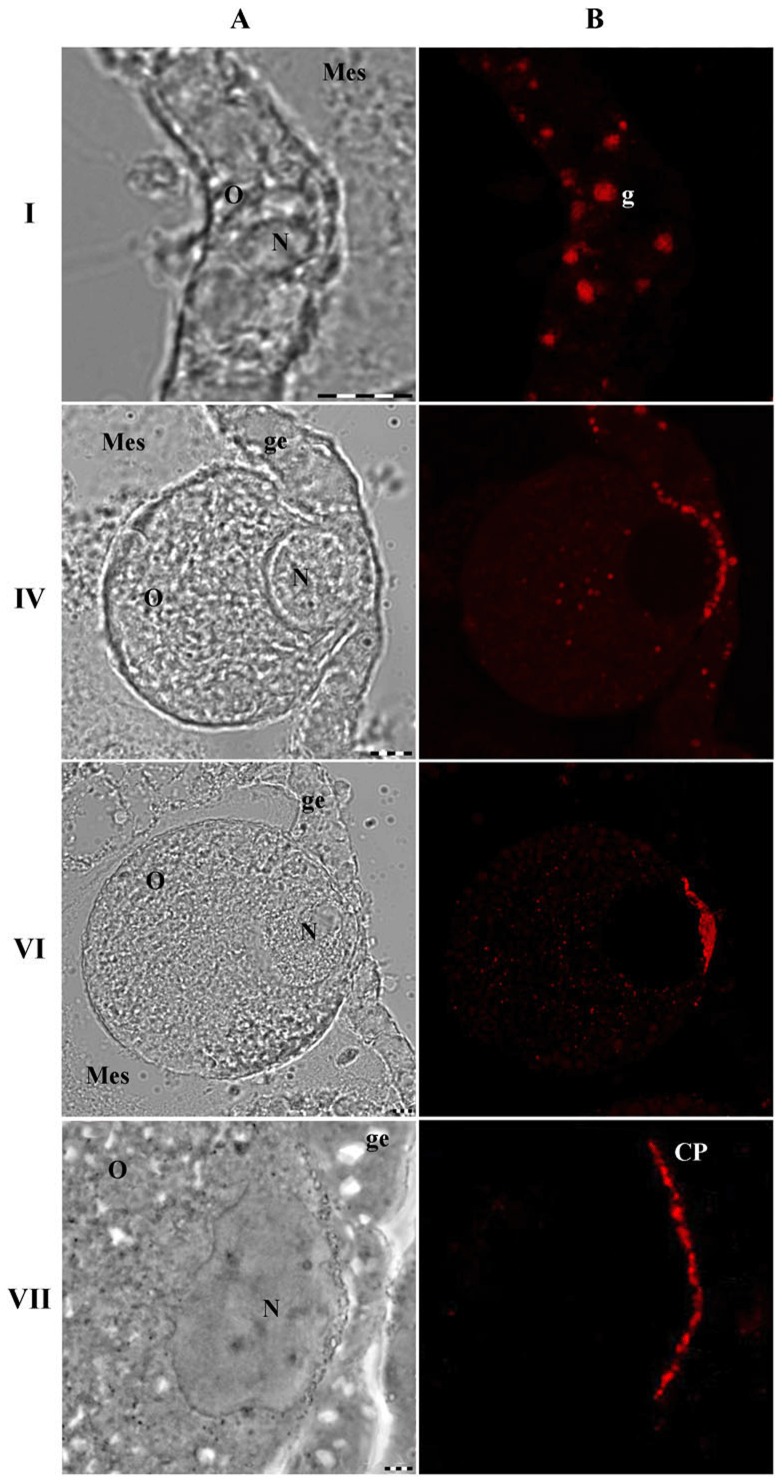
Immunostaining on paraffin sections of medusa female s' gonads. I, IV, VI, VII – development stages of medusa oocyte. First row - phase contrast; second row – immunostaning of the same preparation. Indicated: Mes – adjacent mesoglea, O – oocyte, N – oocytes' nuclei, g – granules in the oocyte; CP – contact plate. For immunostaining RA47 in final dilution 1∶2000; antirabbit AB conjugated with rhodamine in final dilution 1∶200 (Sigma). Scale bar – 10 µm.

Gonad pieces were fixed and processed for immunofluorescence. AB RA47 reveal the plate - place of contact of the germinal epithelium and oocyte on the paraffin sections (Fig. 2). According to its position in between oocyte and germinal epithelium, we named the structure found the “contact plate”.

The description of the *A.aurita* oogenesis is far from being complete. It is known that oocytes arise from the germinal epithelium – the derivate of the bottom wall of the gastric pocket. Some adjacent cells of the germinal epithelium probably take part in the nutrition of the growing oocyte [17]. At late stages, these cells build up funnelshaped cavities in the germinal epithelium just under the mature ova. These cavities serve as cages for spermatizoids [18]. No specific structures have been described in the region of contact between oocyte and adjacent epithelium.

For convenience of description we split oogenesis into 7 stages based upon oocyte size: from 5 µm (stage I) up to 150–170 µm at the end of maturation (stage VII) (Fig. 2). The AB RA47 reactive material appears at the 1^st^ stage as spots dispersed throughout ooplasm (Fig. 2, I). With the oocyte maturation immunoreactive spots collected at the rim of the oocyte and form a lane – the contact plate (Fig. 2, IV–VII)

### Semi-thin sections

Specific characteristics of each stage can be observed on semi-thin sections at the light-optical level. Oocytes arose from a germinal epithelium of endodermal origin consisting of germ cells outpouching within surrounding gastrodermal cells. Both types were spherical cells at the earliest stages of oogenesis.

**Figure 3:**
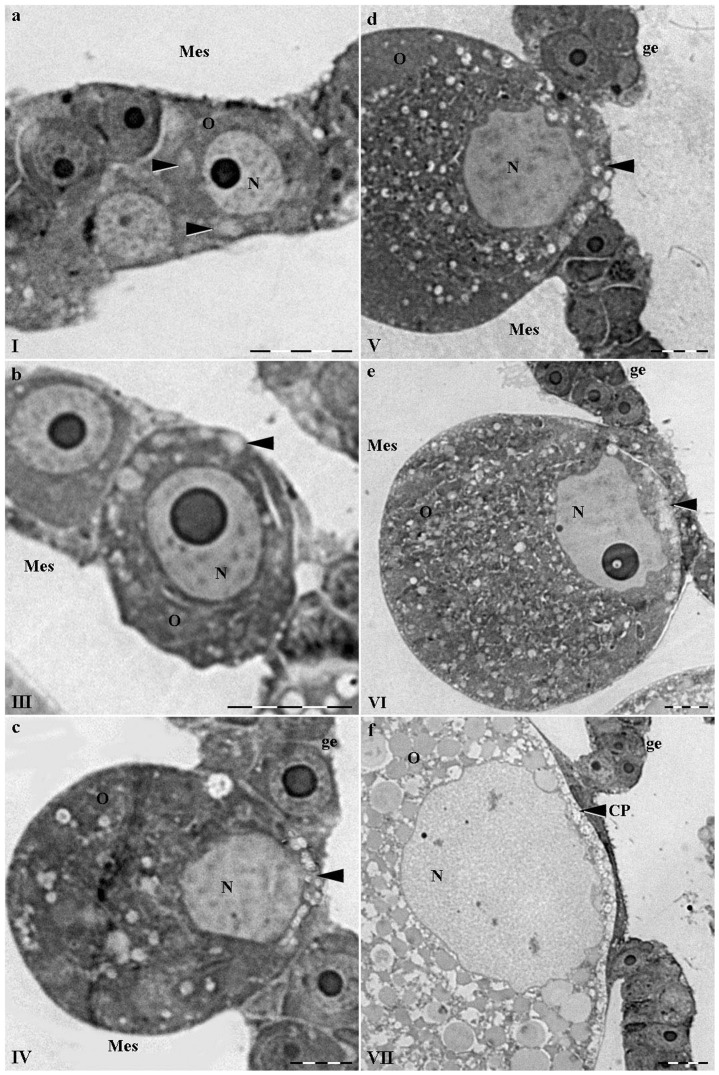
Semi-thin sections of growing oocyte stained with acid fuchsin. I, III, IV, V, VI, VII –stages of medusas' oocyte development; the same structures as on fig. 2 are indicated and in addition ge – germinal epithelium. Oocyte granules and contact plate are indicated with black arrows. Scale bar – 10 µm.

At the I^st^ stage of maturation, the oocyte was almost morphologically indistinguishable from the surrounding cells of germinal epithelium. The oocyte size did not exceed 10–12 µm at the very beginning of its formation (Fig. 3, I). Oocytes could be distinguished by weakly stained granules in the peripheral cytoplasm. The granules, stained with acid fuchsin, were less intense than the surrounding ooplasm (arrows, Fig. 3, I). The nucleus with diameter of about 4–6 µm was located in the oocyte center.

Oocyte diameter increased up to 15–20 µm at the second (II) stage. The nucleus with the diameter of 8–10 µm was still located in the center of the oocyte. With the increased number of weakly stained granules, their position remained the same – dispersed throughout the ooplasm.

At stage III of maturation the oocyte diameter increased up to 25 µm and oocyte began to sink into mesoglea. The nuclear diameter increased up to 10 µm. The nucleus began to shift to the animal pole, where the oocyte continued to be associated with the germinal epithelium cells. The accumulation of granules along the surface of the oocyte was visible (arrow Fig. 3, III). The germinal epithelium at the animal pole of the oocyte began to thin.

At stage IV (oocyte diameter ∼50 µm; nucleus diameter – 20 µm), the oocyte was deep in the surrounding mesoglea. The nucleus was on its definitive position – close to the animal pole in ooplasm. The amount of granules increased and can be distinguished from the newly formed yolk granules. The granules-precursors began to align between nucleus and oocyte membrane (arrow Fig. 3, IV).

At stage V (oocyte diameter - 70–90 µm; nucleus diameter up to 40 µm), the granules could be recognized on the light-optical level, most of them aligned along the germinal epithelium (arrow Fig. 3, V). The expanded nucleus was still at the animal pole and the amount of the yolk granules increased significantly in comparison with the previous stage.

In the last two stages (VI: oocyte diameter 90–130 µm; VII: oocyte diameter 130–170 µm, nucleus diameter up to 60 µm) the fully formed plate between oocyte nucleus and protrusions of the germinal epithelium cells, were visible (arrows Fig. 3, VI, VII). The residual granules of the plate were well distinguished from numerous yolk ones. The plate resembled smooth material surrounded by a granulated material, though at the previous stages, mainly granules were observed. The adult oocytes' cytoplasm was full of 5–7 µm yolk granules.

The dense round structure looking like nucleolus was visible inside the nucleus at all stages of the oocyte maturation. Germinal epithelium underwent changes at the place of oocyte attachment. Its cells, connected with the oocyte, became thinner and lose resemblance to the oocyte precursors from stage II. At the end of maturation, only thin protrusions of the rim epithelium cells retained contact with the oocyte (Fig. 3, VII)

There were glycoproteins with a highly positively charged protein component in the contact plate and in granules – precursors according to the histochemical analysis [19].

### Transmission Electron Microscopy (TEM)

**Figure 4:**
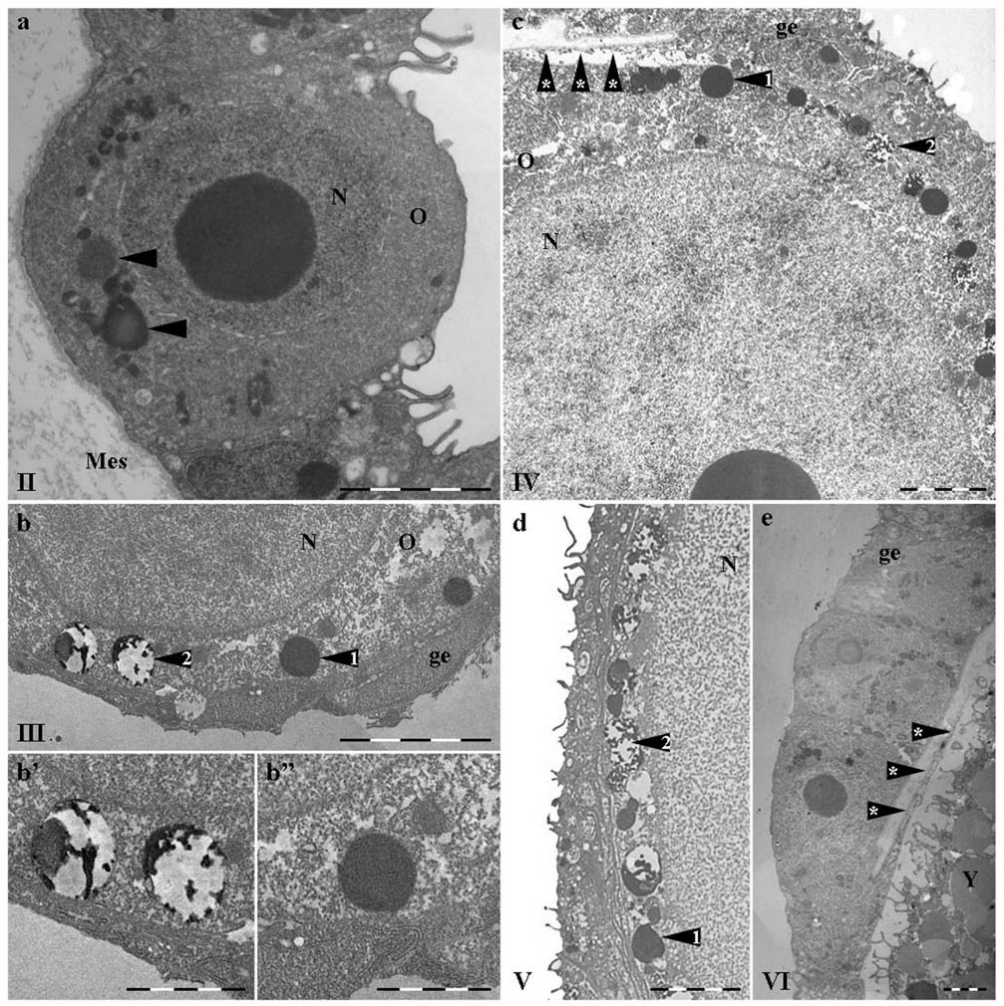
Ultrastructure of medusas' growing oocyte. I, III, IV, VI, VII – stages of medusas' oocyte development; the same structures as on fig. 2 and fig. 3 are indicated and in addition: mt – mitochondrion, Y – yolk granule. Granules are indicated with black arrows and granule type is indicated with 1 and 2 on b (III), c (IV) and d (VI). Germinal epithelium protrusions are indicated by black arrows with an asterisk on c (IV) and e (VI). b′ and b″ represent the high magnification of parts from b (III). Scale bar – 5 µm for a (I), b (III), c (IV), d (VI), e (VI). Scale Bar – 2 µm for b′ (type 2 granule) and b″ (type 1 granule).

The earliest spherical oocytes could be distinguished from surrounding cells of germinal epithelium due to the structural features observed at the electron microscopy level. Most oocytes had nuclei from ∼5 µm in diameter containing a single nucleolus. Their cytoplasm was sparse, containing only free ribosomes, a few mitochondria, and spherical granules of ∼1–2 µm in diameter (arrows Fig. 4, I, a). The granules were dispersed throughout ooplasm. Such a position is in agreement with the one of the AB stained granules (Fig. 2, I).

Two types of granules could be observed from stage III (Fig. 4, III, b′ and b″). One of them was electron-dense and opaque with lamella-like fine bands inside (Fig. 4, III, b″, termed “1”). The other, type 2, was more sparse, nearly transparent, with worm-like ramified electron-dense sticks with breadth of about 30–50 nm. In this case, electron-dense material often abuts to the granules' membrane (arrow 2 at Fig. 4, III, b). Similar bodies were occasionally observed in other cortical regions of the oocytes in vitellogenic oocytes, but they were less common. When oocytes reached 40 µm in diameter, they had moved almost entirely into the mesoglea, but still retained contact with a group of adjacent endodermal germinal epithelium cells.

**Figure 5:**
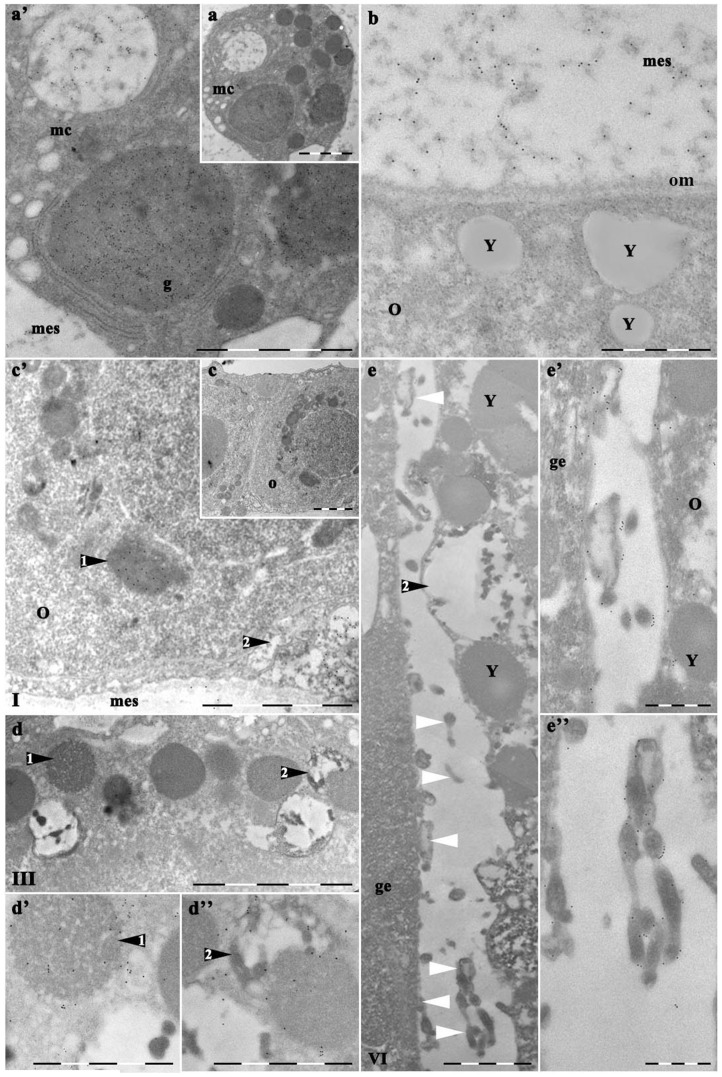
Ultrathin sections of growing oocyte stained with RA47 (immunogold). a - mesogleal cell in adult medusa mesoglea; b – mesoglea embedded vegetative pole of the IV stage oocyte. 10 nm gold-conjugated secondary antibody used (Sigma, final dilution 1∶50). Symbols are the same as on fig. 4 and in addition: mc - mesogleal cell, Y – yolk granules, om – oocytes' basal membrane. White arrows indicate cistern-like structures in the lumen between oocyte and germinal epithelium. Letters with index (i.e. a′, c′ etc) indicate images of the high magnification i.e. parts from images with correspondent letters. Scale bar – 5 µm for a and a′, c and c′, d and e. Scale bar – 2 µm for b, d′ and d″, e′ and e″.

Adjacent cells were distinguished from surrounding germ cells by their flattened appearance and dark-staining qualities. As differentiation continued, the adjacent cells gradually became the only cells in the germinal epithelium in contact with the developing oocyte (Fig. 4, e). The adjacent cells were closely to the contours of the oocyte oolemma, but no specialized junctions were observed between the cells. Intercellular space between the oocyte and germinal epithelium gradually increased from a few nanometers in the early stages of maturation up to 3–7 µm at the final stages (Fig. 4 and 5). Gradually, the adjacent cells became the only cells positioned between the developing oocyte and the subgenital sinus. In the final stages of maturation, adjacent cells with protrusions form a thin plate, which covers about 15% of the surface area of the oocyte (oocyte with a radius of 75 µm: the area covered by the protrusions about 10 000 µm^2^). The germinal epithelium nuclei were positioned laterally at the edge of the plate (Fig. 3, VII), while the remaining cell bodies met in the center, forming tortuous, interdigitating lateral outgrowth (row of arrowheads with white asterisks at Fig. 4, c, e).

At stage IV, granules of both types were aligned in between nucleus and adjacent cells (Fig. 4, c). With the fixed picture TEM gave, it was difficult to get rid of the impression that material of granules 1 underwent processing which makes them acquire the appearance of type 2 granules. At the terminal stages of maturation, few type 2 granules could be found at the apical pole, instead a number of fine vacuolated protrusions 0.1–0.5 µm emerged from the oocyte surface into intercellular gap (Fig. 4, e). These protrusions fill up the intercellular gap together with round vacuolated bubbles 0.4–1 µm in diameter (Fig. 4, e). The narrow zone between the nucleus and oolemma was populated mostly with yolk granules.

### Immunogold

*Mesogleal cells and mesoglea*. The immunogold technique confirmed that mesogleal cells (Mc) synthesize and excrete mesoglein, which is included in fibres. Fibres of ∼40–50 nm are heavily stained with RA47 in mesoglea (Mes, Fig. 5, b) In Mc, most of the label was present in granules of moderate electron density; gold label underlines fine bands inside them. The other type of Mc granules contain fibers very similar in size and appearance to those in Mes, also stained all along (Fig. 5, a′ and b). Again, there is the impression that the material of granules underwent a kind of processing which made them look different.

*Oocyte*. Mature granules of the 1^st^ type of Mc were larger than the oocyte ones in ∼2.5 times (∼5 µm vs ∼2 µm) though oocyte granules 1 were less electron-dense, contained less label; the label was not obviously aligned. The granules of Mc and oocyte type 2 (∼2 µm in diameter) differed even more: the electron-dense labeled material of oocyte granules look more like tubes (40–70 nm wide) than fuzzy fibers inside Mc granules (∼20–40 nm) (Fig. 5, a′ vs Fig. 5, d″). The gold labeling abutted the tubes. A fine smooth fiber sometimes connected tubes inside oocyte granules 2 (Fig. 5, d″).

At stage IV, mesoglea surrounded the oocytes' vegetable pole (Fig. 5, b). Only fibers in Mes were heavily stained with AB; no label was observed in this part of the oocyte. Young yolk granules had blurred boundaries with tubes of the endoplasmic reticulum close to them. The absence of any label inside oocyte and the relatively smooth appearance of the oolemma, without obvious vesicles (blebs) coming from the outside, argue against heavy uptake of the nutrient from Mes in this part of the oocyte. In spite of the question of the yolk granules origin, they never took label of RA47 AB and were distinguished from 1 and 2 (specific) granules at any stages of vitellogenesis.

A remarkable difference was observed between oocyte animal and vegetable poles from stage III. The animal poles' feature was the line of granules of 1 and 2 types between nucleus and oolemma. This line had a distinct border; little precursors of the yolk granules were visible in the ooplasm (Fig. 3, e). Yolk granules were characteristic for the vegetable pole deep in the mesoglea. The vegetable pole oocyte surface was covered with unevenly spaced, irregular fine microvilli (Fig. 5, b).

The animal pole looked quite different at the late stages of the oocyte maturation. Mostly yolk granules fill the ooplasm. These granules differed from both granules 1 and 2: they were larger, more electron-dense bodies ∼5 µm in diameter; with smooth content and smooth membrane, often with the whitening in the core (Fig. 5, e). The contact plate was formed from the granules 1 and 2 material in the extracellular space at the animal pole, but no such granules remained at the vegetable pole (Fig. 5, b).

At the late stages of vitellogenesis, some material similar in appearance to that from granules 2 was dispersed along the gap lumen (Fig. 5, e, white arrows) and it was these patches that were outlined with the label (Fig. 5, e′ and e″). Probably, the electron-dense core of tube-like structures was not accessible to the AB, though some kind of vacuolization was noted inside them.

**Figure 6:**
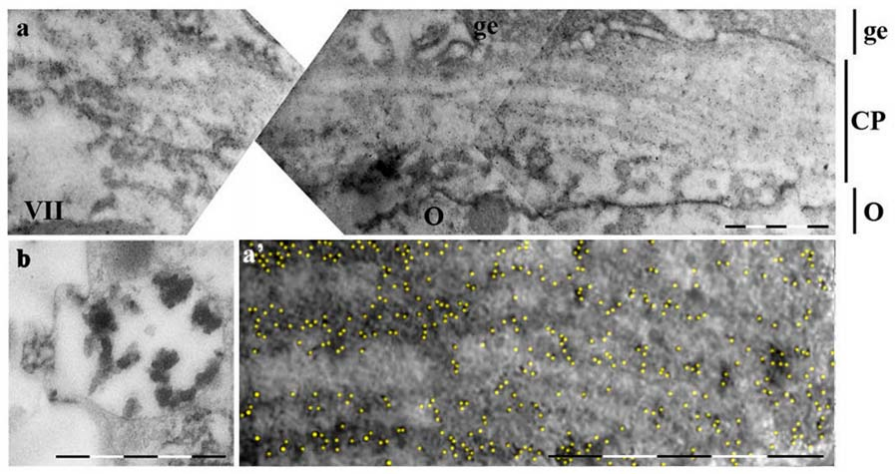
Fully formed contact plate immunogold stained. Staining conditions are the same as on Fig. 5. a –contact plate of the oocyte VII final stage at low magnification. Indicated with lines at the right are: O – oocyte, CP – contact plate, ge – germinal epithelium. Scale bar –5 µm. a′ – high magnification of the part of the image on a; gold particles pseudocolored yellow. Scale bar–2 µm. b – type 2 granule presumably in the process of excretion (not stained). Scale bar – 2 µm.

The thickness of the contact plate was 5–7 µm at the widest point at the last stage of oocyte maturation (Fig. 6, a). Two zones could be distinguished inside it: the fibrillar zone close to the adjacent cells of the germinal epithelium and the intermingling zone close to the oocyte. When one was lucky to observe the excretion of the tubular-like patches from granule 2, it was visible that electron-dense material underwent vacuolization outside the granule membrane, became less dense and more fibrillar (Fig. 6, b). An intermingling zone made up of such vaculolated tubes gradually turned into fiber bundles. The label became heavier and aligned with the fiber formation from the vacuolated patches (Fig. 6, a′). The fully formed fiber was 10–20 nm thick, labeled all along and often doubled\multiplied to form bundles. The bundles in contact plate were aligned parallel to each other and to the oocyte surface.

### *In vitro* fertilization

**Figure 7:**
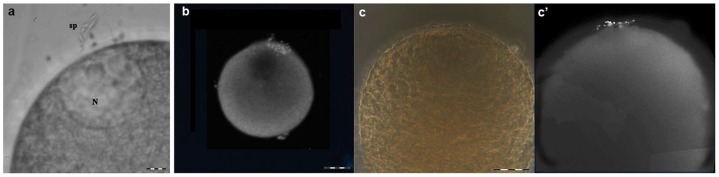
Fertilization *in vitro*. Spermatozoids added to medusa eggs *in vitro* in sea water. a - phase contrast microscope, squash preparation. N – eggs' nucleus, sp – spermatozoids. Scale bar – 10 µm. b – c′ – whole egg in the process of fertilization (non-squashed); b – DAPI staining in grayscale, low magnification; c and c′ - phase contrast and DAPI fluorescence in grayscale, the same field. Scale bar – 50 µm.

At the terminal stage of oogenesis the oocytes' contact with the germinal epithelium was very weak and they were released into the sea water mechanically by pipetting. *In vitro* fertilization was done on slide in sea water with the spermatozoids added. Spermatozoids went only to the contact plate during the eggs fertilization (Fig. 7, a). It was visible on DAPI stained material, that the rest of the egg membrane (oolemma) did not attract spermatozoids (Fig. 7, b–c). DAPI brightly stained spermatoziods formed a kind of the cap on eggs' contact plate (Fig. 7, c′). It was likely that the contact plate located on the animal pole promoted the male and female pronuclei spatial association.

A mucous envelope was absent around the oocytes, which were fertilized even in the genital sinus [20]. The ZP-containing contact plate marked and narrowed the place for the spermatozoid to go: the nucleus was located close to the contact plate and the oocyte was aligned in the germinal layer so as to expose the contact plate in the direction of the water flow [21]. The high *M_r_* of the contact plates' ZP-proteins made them sufficient to contain the domain for spermatozoid recognition.

## Discussion

### The contact plate was mistaken as a layer of yolk granules

The structure similar to the contact plate was observed earlier [17], but the authors paid most attention to vitellogenesis. Their ultrastructural data suggested that different types or classes of yolk precursors enter the oocyte through the trophocytes and via the surrounding mesoglea. The structure was clearly visible and marked in their paper at the semi-thin sections. Following the logic of the paper, the authors claimed that the material of the structure was the small granules of yolk precursors provided by trophocytes. Our histochemical data and immunostaning evidence shows that granules-precursors of the contact plate, differ from the surrounding yolk granules (Fig. 2, Fig. 6) [19]. The internal position of the granules at early stages of oogenesis and the position of the plate itself led us to the suggestion that trophocytes could be involved in the synthesis of their material only to the extend of providing liquid nutritions. We never observed prominent coated vesicles around any oocyte granules. Instead of coated vesicles prominent endoplasmic reticulum (ER) surround granules at early stages and a kind of contact could be traced between granules and ER (Fig. 4, b′ and b″, Fig. 5, d′). Yolk granules were also likely to be synthesized by oocyte itself: acridine orange, which revealed RNA [22], stains ooplasm homogeneously at early stages (I–III) but RNA were concentrated around young yolk granules from stage IV [19]. Young yolk granules were also surrounded with endoplasmic reticulum which sometimes has the appearance of bubbles (Fig. 5, b). No well defined egg envelope or extracellular coat was observed in accordance with the data previously described [17].

### Mesoglein AB and comparison with mesoglein

Instead of the egg coat, we found extracellular structure, the contact plate, which resembles mammalian Zona pellucida. Our main instrument was RA47 AB against mesoglein. ZP-domain occupies about half of the whole amino acid sequence of the mesoglein [8]. Antibodies RA47 could be directed against ZP-domain, at least against part of it. If so, ZP domain containing proteins are definitely members of the contact plate. Genomes of most animals available in Databases contain more than one protein of ZP family.

Dataset of the published invertebrate genomes (http://www.ncbi.nlm.nih.gov/genomes/leuks.cgi) was used for the search of proteins with similarity to mesoglein by conventional programs (Blast, ClustalX; Philyp-3.69; GSview 4.6); only ZP-domain containing proteins (determined by SMART), both described and predicted as ORFs, were taken into consideration; the filter was set to e-Value less than e^−10^ in order to cut off false positive. The automatically built unrooted cladogram reveals 52 proteins, all of them but mesoglein were predicted. The closest mesoglein relative was predicted Hydra protein of similar length (H.mag XP_002159562); it does not contain any other domains than ZP. Hydra proteins recognised by RA47 AB [6], provide evidence that our AB are directed against ZP domain.

Contact plate proteins are recognized by RA47 AB (1) on paraffin sections (Fig. 3); (2) in immune-gold TEM (Fig. 5,6); (3) on immunoblot after denaturing condition of SDS-PAGE (Fig. 1, I); (4) on immunoblot after AU-PAGE (Fig. 1, II). AU-PAGE is sensitive to the proper charge of the proteins: only positively charged proteins, such as histones, could be separated [12]. The titration curve deduced from the mesoglein aa sequence shows pI 9.03 and charge 30 (calculated using an EMBOSS IEP tool) at pH 7.5 in agreement with experimental data. The high positive charge of mesoglein is unlike that of most other ZP domain containing proteins. Only 12.5% of ZP domain proteins predicted pI higher than pI 8. The mean predicted pI value of ZP domain-containing proteins is pI 6.3 and the mean pI of ZP domains is pI 6.2. ZP protein(s) of *A.aurita* gonads are also charged, though they possess lower mobility than mesoglein itself or its precursors from mesogleal cells (Fig. 1, II).

There could be two possible explanations for the difference between the contact plates' proteins and mesogleins' presursors in *M_r_* and charge. The first is: the post-translational moidifications are a characteristic feature of the ZP-domain family of proteins [23]. Mesoglein itself undergoes modifications when it incorporates into mesoglea. The second interpretation is that the contact plates' proteins might be other than mesoglein representatives of the ZP family. Only future experimental work will allow us to examine these possibilities.

### ZP proteins processing

Contact plate material appears in granules, which are fused into plate later on. This is typical for ZP-proteins. Extracellular proteins that assemble into filaments or matrices have evolved a variety of strategies to regulate their assembly both spatially and temporally. These proteins should not polymerize prematurely inside cells. There is evidence that regulation of ZP domain protein polymerization is achieved through a relatively complex, mutually dependent set of events. Nearly all precursors of ZP domain proteins are characterized by either a C-terminal TMD or GPI-anchor. However, in all cases the C-terminal propeptides are lost, either before or during protein polymerization, by proteolytic cleavage at conserved basic sites located immediately after the ZP domain [23]. The cloning of the mesoglein precursor and ZP protein from female gonads will allow us to clarify the question. Right now, we assume that the appearance of ZP protein in the form of granules is the preparation for the future excretion and fusion into contact plate. Probably, the two types of morphologically distinct granules reflect a part of the set of events, which lead to the ZP protein polymerization into contact plate fibers outside oocyte.

### Filaments

The fully formed contact plate was up to 10 µm thick and it occupied about 15% of the oocyte surface. In linear dimension, the contact plate length was ∼130–150 µm while oocyte diameter is of the same order. Its filament content was obvious at the fully mature oocyte and these very filaments possess the heaviest label among all the oocyte preparations, suggesting that ZP domain was exposed to the AB47 (Fig. 6, a and a′). Contact plate fibers are of the same order as the fine fibers of mesoglea (20–50 nm)

Mammalian Zona Pellucida is relatively well investigated at the ultrastructural level. Different families of ZP domain proteins assemble into similar supramolecular structures. Zona Pellucida fibers of mouse, hamster [24], rat [25], [26], [27], human [28], guinea pig [29] are of the same order – 10–20 nm as well as the size of filaments reconstructed from the purified proteins – 10–15 nm [23]. In case of medusa contact plate, we observed the filaments of the similar size collected in bundles (Fig. 6, a′)

The parallel alignment of fibers to each other and to the egg surface differed from the 3D meshwork observed in mammalian Zona. Probably, this happens due to the lack of the whole set of ZP proteins involved in contact plate formation. No less than 3 ZP proteins produce the full scale Zona in high mammals. Artificial fibers made up of one ZP protein produce aggregates of long fibrils as it is visible in the contact plate (Fig. 6 a) [23]. The specific architecture of the contact plate may arise from combining a common building block of proteins - the ZP domain itself, and additional sequences with specific biological functions. The *M_r_* of contact plate ZP protein gives place to other domains probably involved in fertilization.

Very little is known about the constituents of the invertebrate eggs' ECM. Only a handful of proteins have been isolated and characterized – primarily from ascidians, sea urchins, bivalves, and the dipterian Drosophila - and these proteins bear little resemblance to one another or the vertebrate ZP proteins. The same is true for the ZP-containing predicted ORFs [21].

The structure of an invertebrate egg ECM is more variable than its vertebrate analogue. One reason for this diversity may lie in much greater diversity of reproductive methods used by invertebrates, in spite of many of them being aquatic animals. The wide evolutionary distance separating these animals from diverged clade of vertebrates and invertebrate clades themselves from each other, also adds to the ECM diversity. Although a vertebrates' egg ECM is a series of glycoprotein shells that surround the egg, those of invertebrates prove more complex [10]

Most of the eggs' ECM covers the whole egg. There are very few examples of the egg polarity reflected in the polarity of egg ECM. A regional specialization of the egg ECM is ubiquitous in dipterian insects, whose eggshell is specifically molded for sperm entry only at the anterior pole [30], [31], [32]. The contact plate, with its 15% coverage of the egg surface, is a unique structure, which could be the first sign of the future Zona Pellucida.

The contact plate of *A.aurita* oocyte and egg marks its animal pole and resembles Zona Pellucida in the following features: (1) it attracts spermatozoids; (2) the material of the contact plate is synthesized by oocyte and stored in granules; (3) these granules and contact plate itself contain ZP domain protein(s); (4) the contact plate is an extracellular structure made of fiber bundles similar to those of ZP proteins. Future investigations will clarify its protein content.
